# Almost 10 years of PET/MR attenuation correction: the effect on lesion quantification with PSMA: clinical evaluation on 200 prostate cancer patients

**DOI:** 10.1007/s00259-020-04957-x

**Published:** 2020-07-28

**Authors:** Borjana Bogdanovic, Andrei Gafita, Sylvia Schachoff, Matthias Eiber, Jorge Cabello, Wolfgang A. Weber, Stephan G. Nekolla

**Affiliations:** Technical University of Munich, School of Medicine, Klinikum rechts der Isar, Department of Nuclear Medicine, Munich, Germany

**Keywords:** Whole-body PET/MR, Attenuation correction, PET quantification, Prostate cancer, Bone atlas, Truncation correction

## Abstract

**Purpose:**

After a decade of PET/MR, the case of attenuation correction (AC) remains open. The initial four-compartment (air, water, fat, soft tissue) Dixon-based AC scheme has since been expanded with several features, the latest being MR field-of-view extension and a bone atlas. As this potentially changes quantification, we evaluated the impact of these features in PET AC in prostate cancer patients.

**Methods:**

Two hundred prostate cancer patients were examined with either ^18^F- or ^68^Ga-prostate-specific membrane antigen (PSMA) PET/MR. Qualitative and quantitative analysis (SUV_mean_, SUV_max_, correlation, and statistical significance) was performed on images reconstructed using different AC schemes: Dixon, Dixon+MLAA, Dixon+HUGE, and Dixon+HUGE+bones for ^18^F-PSMA data; Dixon and Dixon+bones for ^68^Ga-PSMA data. Uptakes were compared using linear regression against standard Dixon.

**Results:**

High correlation and no visually perceivable differences between all evaluated methods (*r* > 0.996) were found. The mean relative difference in lesion uptake of ^18^F-PSMA and ^68^Ga-PSMA remained, respectively, within 4% and 3% in soft tissue, and within 10% and 9% in bones for all evaluated methods. Bone registration errors were detected, causing mean uptake change of 5% in affected lesions.

**Conclusions:**

Based on these results and the encountered bone atlas registration inaccuracy, we deduce that including bones and extending the MR field-of-view did not introduce clinically significant differences in PSMA diagnostic accuracy and tracer uptake quantification in prostate cancer pelvic lesions, facilitating the analysis of serial studies respectively. However, in the absence of ground truth data, we advise against atlas-based methods when comparing serial scans for bone lesions.

## Introduction

Integrating positron emission tomography (PET) and magnetic resonance imaging (MR) with the aim of combining the best assets of both modalities into a “one-stop-shop” introduced technical and logistical challenges. PET attenuation correction (AC) has been a thorn in the side of PET/MR systems for almost a decade due to the absence of a direct and definitive method for calculating an attenuation map. The technical obstacles of coupling a radioactive transmission source or an X-Ray tube to a PET/MR system occur as a result of the presence of a strong *B*_0_ magnetic field, as well as its inhomogeneity and the gradient-field nonlinearities already present in the relatively narrow PET/MR bore size. Additionally, a radioactive transmission source, given that traditional 511 keV transmission scans need long acquisitions, would make already long PET/MR acquisitions furthermore cumbersome. All this eliminates the possibility of a conventional transmission scan. Attenuation maps cannot be directly derived from the second modality either, as the conventional MR signal provides information on relaxation times and ^1^H proton densities, which, unlike electron densities (as it is the case with computed tomography, i.e., CT in PET/CT), cannot be directly converted or extrapolated to attenuation coefficients for 511-keV photons. Hence, the PET/MR systems need an alternative PET AC method to ensure high accuracy and enable PET image quantitation.

In the clinical routine, this alternative PET AC method is usually based on a Dixon MR sequence, owing to its time-efficient acquisition, straightforward implementation, and availability right from the start of PET/MR systems. The standard Dixon method generates separate fat and water images, and then, using thresholding, morphological image processing, and connected component analysis, it allows data segmentation into four compartments (fat, lung, soft tissue, and background air), which are subsequently assigned the corresponding linear attenuation coefficients (LACs). Bone is not accounted for, as bone segmentation, especially outside the brain, gets complicated using conventional MR sequences. The complications arise as a result of the rapidly decaying MR signal from cortical bone due to the short T2 relaxation time and can get worse due to the presence of the signal created by cancellous bone. In brain imaging, the issue of bone segmentation is often addressed by employing special pulse sequences with ultra-short echo times (UTE) to collect the signal from bone [1]. Recently, sequences with “zero” echo times (ZTE) have been suggested for this purpose [2]. Unfortunately, due to a low signal-to-noise ratio (SNR), these pulse sequences need longer acquisitions and are less time-efficient for multiple bed positions in clinical whole-body imaging. In addition, they tend to be affected by magnetic field inhomogeneity and their accuracy declines close to bone/air interfaces.

Consequently, in whole-body imaging, bone is usually treated like soft tissue and assigned a LAC accordingly. The effects of this approach on lesion quantification have been examined in different settings by a few groups working with ^18^F-FDG PET data and using CT-based attenuation maps as reference [3–6], one of which produced a simulation [7]. The clinical interpretations for the involved patients were collectively deemed unaffected until a new way of including bone in PET AC was suggested in the form of an offline-constructed bone model that is to be registered to the Dixon attenuation map and consists of major bones only (skull, spine with sacrum, left and right hip, left and right femur) [8]. After this method had become a part of the PET/MR clinical routine, some supportive results with ^18^F-FDG PET data [9], as well as some less convincing results with ^18^F-fluciclovine [10], followed, leaving the problem status somewhat inconclusive.

Another technical challenge of the standard Dixon AC method known to the PET/MR community has been the truncations in the transverse plane of the MR-based attenuation maps beyond the field of view (FoV) of approximately 50 cm [11]. This constraint of the MR FoV in all geometric directions emerges as a result of the inhomogeneity of the main magnetic field *B*_0_ as well as the gradient-field nonlinearities, limiting the spatial encoding accuracy and leading to geometrical distortions (experienced as image truncations) in the distal part of the FoV, where usually the patient’s arms are positioned. Considering that the PET FoV can be as wide as 60 cm, one possible approach has been a maximum-likelihood algorithm serving to estimate the missing voxels of the truncated attenuation maps based on the attenuated PET emission data, known as maximum-likelihood reconstruction of attenuation and activity (MLAA) [12].

The latest approach offered by the system analyzed in this study uses gradient enhancement (homogenization using gradient enhancement or HUGE) to find the optimal readout gradient field needed to compensate the *B*_0_ inhomogeneity as well as gradient nonlinearities and thus extend the MR FoV [13]. In practice, each slice position (where arms are present) is to be measured twice using the optimal readout gradient amplitude for the left and right arm, respectively. The image truncations are hence avoided.

The motivation for addressing PET/MR image accuracy and quantitation with these various AC techniques developed over the years has been their application in oncology, including both diagnostics and treatment monitoring. One important application for PET/MR is prostate cancer (PCa) imaging [14, 15]. For the purposes of PCa localization, e.g., PET/MR holds an advantage over both multiparametric MRI (mpMRI) and PET imaging alone [16]. Currently, ligands of the prostate-specific membrane antigen (PSMA) are the most promising PET tracer for PCa imaging [17] and both ^68^Ga- and ^18^F-labeled PSMA ligands are under clinical evaluation.

The aim of our analysis was to link the technical to the clinical aspects by qualitatively and quantitatively evaluating the impact the two approaches of extending the MR FoV and including the offline-constructed bone model in the PET attenuation map have on lesion quantification in prostate cancer patients. Furthermore, our goal was to evaluate how this variety of currently available AC schemes are reflecting on serial studies and the “procedural” stability and intracomparability of the results. Thus, we performed a retrospective analysis of 200 PCa patients examined with ^18^F- and ^68^Ga-labeled PSMA ligands using a PET/MR system.

## Materials and methods

### Study protocol

Having had access to two important clinical radiotracers for PCa, ^18^F-PSMA and ^68^Ga-PSMA, we included both in our study to demonstrate the effects of different AC schemes with two different radioisotopes, featuring two distinctive clearance routes, differences in high-uptake structures, and thus scatter effects. Two hundred male patients, aged 68 ± 9 years, with a mean BMI of 26 (min = 18.7, max = 32.9), diagnosed with primary or recurrent prostate cancer were included in this retrospective study. Among these, 116 patients were injected with 317 ± 47 MBq of ^18^F-PSMA, while 84 patients received 109 ± 15 MBq of ^68^Ga-PSMA. All patients had lesions in the pelvis and underwent simultaneous PET/MR whole-body examinations 80 min p.i. (^18^F-PSMA) or 60 min p.i. (^68^Ga-PSMA) with a clinical 3T PET/MR hybrid system (Biograph mMR, Siemens Healthcare, Erlangen, Germany) [18]. The PET/MR AC acquisitions were performed with CAIPIRINHA parallel imaging [19] in 4–5 bed positions, using the head/neck coil, the spine array coil, and the flexible body matrix coils covering the FoV. All patients signed a written consent for evaluation of their data, and the institutional review board of the Technical University Munich (permit 5665/13 for ^68^Ga-PSMA and permit 257/18S for ^18^F-PSMA) approved this analysis.

### PET image reconstruction

The acquired images were reconstructed with the standard console reconstruction tool (RetroRecon card) in the PET/MR system using ordinary Poisson ordered-subset-expectation maximization (OP-OSEM) iterative reconstruction algorithm with 3 iterations, 21 subsets, matrix size 344 × 344, zoom 1, and a 4-mm FWHM Gaussian smoothing kernel. Relative scatter correction was applied with the exception of cases with significant halo artifacts around the bladder, where absolute scatter correction was used with a maximum scatter fraction of 40% [20]. Exclusively for ^68^Ga-PSMA studies, it was combined with the prompt gamma correction [21, 22].

### Attenuation correction

#### AC schemes with the ^18^F-PSMA PET dataset

All ^18^F-PSMA PET scans took place after the system software update to version *syngo* MR E11 (Siemens Healthcare Erlangen, Germany). The images were corrected for attenuation using the volumetric interpolated breath-hold examination (VIBE) with four different attenuation map generation techniques available with this update: standard Dixon-VIBE fat-water separation technique, Dixon-VIBE extended with MLAA, Dixon-VIBE extended only with HUGE, and Dixon-VIBE extended with both HUGE and the offline-constructed bone model.

#### AC schemes with the ^68^Ga-PSMA PET dataset

The ^68^Ga-PSMA PET images, acquired predominantly before the update, were corrected for attenuation using only two different attenuation map generation techniques: standard Dixon-VIBE and Dixon-VIBE extended with the offline-constructed bone model. The extension with HUGE was impossible as it required additional MR acquisitions with gradient enhancement, unavailable prior to the update. However, we aimed for a larger cohort and decided to include them due to the more widely used isotope.

### PET image analysis

An experienced nuclear medicine specialist, who also identified all PCa lesions in the pelvis, qualitatively evaluated the reconstructed PET images. For a quantitative assessment, we analyzed the SUV_mean_ and SUV_max_ for each detected lesion located in the entire pelvis, as well as the SUV_mean_ of its respective background (BG), using the PERCIST reference volumes of interest with commercial software (syngo TrueD, Siemens Healthcare, Erlangen, Germany).

In the absence of absolute ground truth data, the results were compared using linear regression against standard Dixon-VIBE, which was available on the system from the very beginning. Additionally, the Wilcoxon signed-rank tests were performed to test whether the SUV_mean_ and SUV_max_ values in PET images reconstructed using each AC method were significantly different from the corresponding values in the PET images reconstructed with other evaluated methods. We investigated variance and correlation between the methods and calculated the coefficient of correlation *r* as well as the coefficient of determination *R*^2^ (“*R* squared”) for each evaluated method against standard Dixon-VIBE. The differences between the AC methods, i.e., the estimated bias and fluctuations in SUV_max_, were additionally visualized and evaluated using the Bland-Altman plots (the agreement limits were defined by the 96% confidence level).

## Results

### Procedural failure

The MR FoV extension with HUGE failed in total once, leaving one arm uncorrected for truncation for unknown reasons. The offline-constructed bone model registration failed in total 8 times (4% of all cases), whereby 4 times, the spine was missing, leaving the pelvis unaffected, while the other 4 times, the pelvis was missing: 3 times due to the presence of metallic femur/hip implants and once randomly. The four affected pelvises were missing both hips and femur bones. These cases were thus excluded from the following analyses, leaving the number of analyzed scans at 196.

### Qualitative assessment

In the 113 ^18^F-PSMA scans, 175 out of 262 lesions were found in the soft tissue of the pelvis (intraprostatic lesions in the prostate, local recurrence, pelvic lymph node metastases) and 87 in the bones (pelvic bones and proximal femur). The average number of analyzed pelvic soft tissue lesions per patient was 1.7 (min = 1, max = 7); the average number of analyzed osseous pelvic lesions per patient with osseous metastases was 4.4 (min = 1, max = 9).

In the 83 ^68^Ga-PSMA scans, 102 out of 129 lesions were detected in the pelvic soft tissue and 27 lesions in the bones (pelvic bones and proximal femur). The average number of analyzed pelvic soft tissue lesions per patient was 1.6 (min = 1, max = 8); the average number of analyzed osseous pelvic lesions per patient with osseous metastases was 1.9 (min = 1, max = 5).

**Fig. 1:**
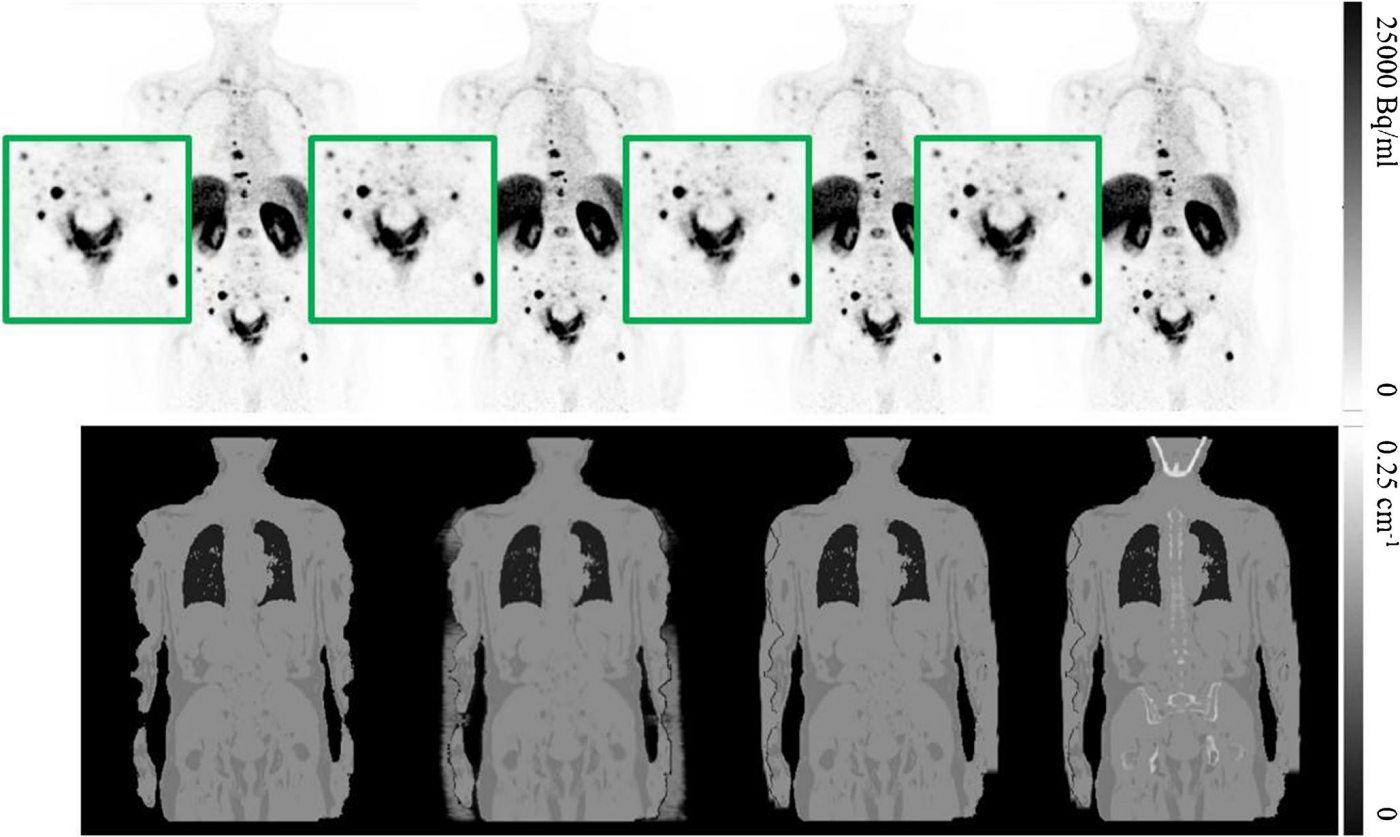
Four reconstructed ^18^F-PSMA PET images (top) with the corresponding four attenuation maps (bottom), left to right: standard Dixon-VIBE, Dixon with MLAA, Dixon with HUGE, and Dixon with HUGE and bones

**Fig. 2:**
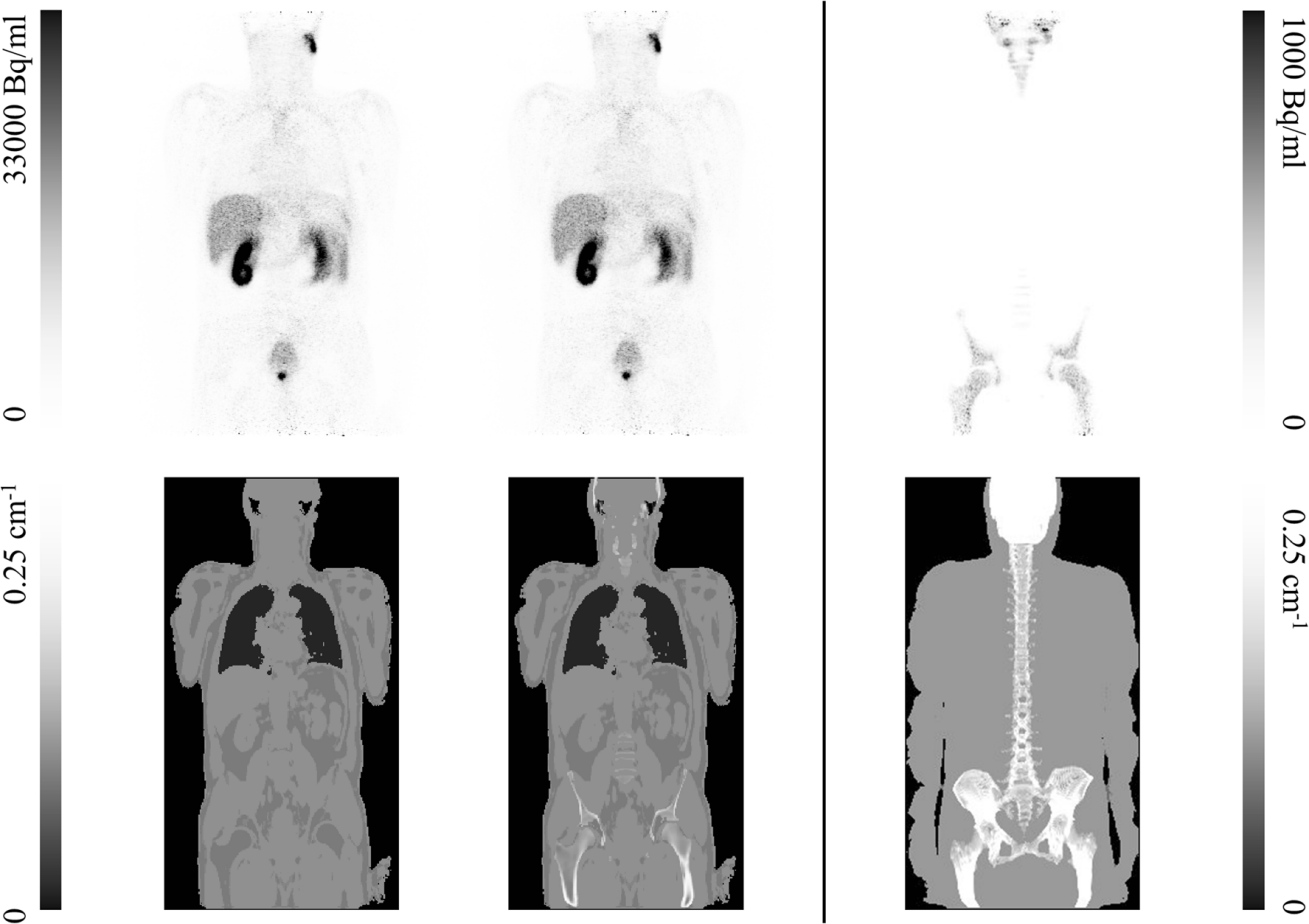
Two reconstructed ^68^Ga-PSMA PET images: standard Dixon-VIBE (top left) and Dixon-VIBE with bones (top middle) with the two corresponding attenuation maps (bottom left and middle, respectively), the difference between the two PET images (top right), and the maximum intensity projection of the Dixon attenuation map obtained with HUGE and atlas bones (bottom right)

The qualitative assessment of both ^18^F-PSMA and ^68^Ga-PSMA datasets showed no visually perceivable differences influencing diagnostic accuracy between the corresponding images reconstructed with the analyzed methods. No new lesions were detected with any of the alternative AC schemes with respect to standard Dixon. An example of the four AC schemes assessed with ^18^F-PSMA is seen in Fig. 1, together with the corresponding reconstructed PET images. Equivalently, an example of the two AC schemes assessed with ^68^Ga-PSMA is seen in Fig. 2, together with the corresponding reconstructed PET images and their difference map, as well as the maximum intensity projection of the Dixon AC scheme with HUGE and bones.

### Quantitative assessment

Within the ^18^F-PSMA datasets, using the standard Dixon-VIBE method as reference, mean relative difference (MRD) was calculated, as well as minimum and maximum relative differences. The results for soft tissue and bone lesions are shown in Tables 1 and 2, respectively. Upon testing all three AC schemes against standard Dixon in the soft tissue, the Wilcoxon signed-rank test showed statistical significance (*p*_max_ < 0.001) in both the SUV_mean_ and SUV_max_ differences between the corresponding images reconstructed using the evaluated methods. The corresponding background uptake also showed significant differences between the evaluated methods (*p*_max_ = 0.048). In the bones, the results acquired followed the soft tissue ones, yielding *p*_max_ < 0.001 for both SUV_mean_ and SUV_max_, and *p*_max_ = 0.02 for the background uptakes.

**Table 1:** Lesion uptake in soft tissue−−^18^F-PSMA PET

Soft tissue	HUGE w/ bone vs. Dixon				
	MRD	[MIN, MAX]	*r*	*R*^*2*^	*p* value
SUV_max_	3.91 ± 2.50	[− 1.13, 11.75]	0.9993	0.9924	< 0.001
SUV_mean_	3.84 ± 2.73	[− 5.90, 12.18]	0.9992	0.9921	< 0.001
BG	-	-	0.8971	0.5407	< 0.001
Soft tissue	HUGE w/o bone vs. Dixon				
	MRD	[MIN, MAX]	*r*	*R*^*2*^	*p* value
SUV_max_	1.91 ± 1.89	[− 4.78, 8.11]	0.9996	0.9978	< 0.001
SUV_mean_	1.85 ± 2.01	[− 6.58, 8.65]	0.9995	0.9979	< 0.001
BG	-	-	0.9323	0.8159	< 0.001
Soft tissue	MLAA vs. Dixon				
	MRD	[MIN, MAX]	*r*	*R*^*2*^	*p* value
SUV_max_	4.61 ± 2.88	[− 1.57, 19.13]	0.9993	0.9919	< 0.001
SUV_mean_	4.47 ± 3.14	[− 4.08, 20.56]	0.9991	0.9922	< 0.001
BG	-	-	0.8881	0.5830	0.048

**Table 2:** Lesion uptake in bones−−^18^F-PSMA PET

Bone	HUGE w/ bone vs. Dixon				
	MRD	[MIN, MAX]	*r*	*R*^*2*^	*p* value
SUV_max_	10.02 ± 5.20	[− 1.95, 26.92]	0.9984	0.9780	< 0.001
SUV_mean_	10.22 ± 5.25	[− 0.86, 28.18]	0.9983	0.9791	< 0.001
BG	-	-	0.9512	0.7749	< 0.001
Bone	HUGE w/o bone vs. Dixon				
	MRD	[MIN, MAX]	*r*	*R*^*2*^	*p* value
SUV_max_	2.77 ± 3.11	[− 5.35, 15.65]	0.9996	0.9975	< 0.001
SUV_mean_	2.75 ± 3.05	[− 3.09, 15.61]	0.9997	0.9978	< 0.001
BG	-	-	0.9716	0.8792	< 0.001
Bone	MLAA vs. Dixon				
	MRD	[MIN, MAX]	*r*	*R*^*2*^	*p* value
SUV_max_	5.73 ± 4.45	[− 7.05, 23.02]	0.9994	0.9918	< 0.001
SUV_mean_	5.73 ± 4.27	[− 4.20, 23.12]	0.9994	0.9926	< 0.001
BG	-	-	0.9249	0.6254	0.02

**Fig. 3:**
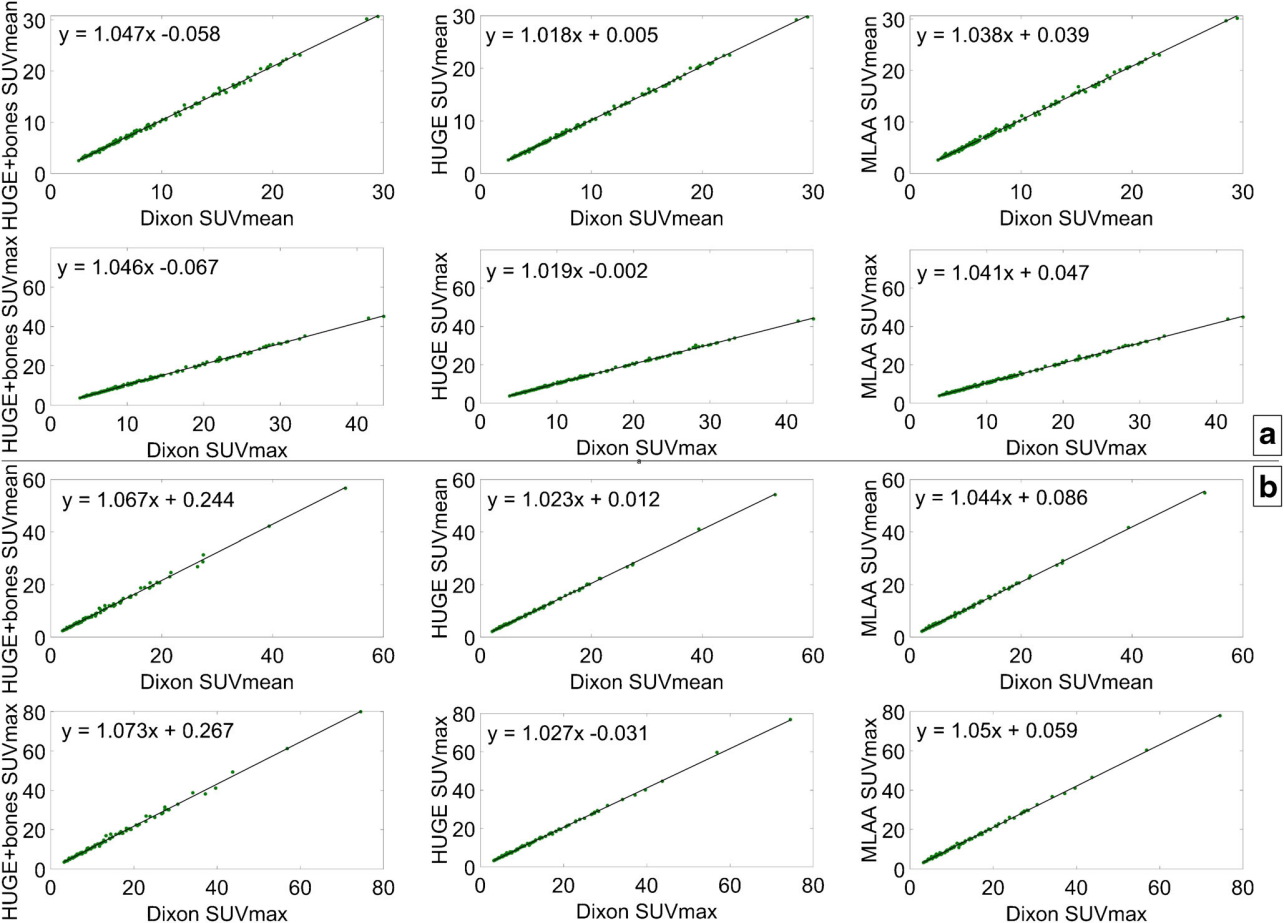
Correlation between the SUV_mean_ and SUV_max_ computed from the data reconstructed with standard Dixon and each of the three evaluated AC methods in soft tissue (**a**) and bones (**b**)

**Fig. 4:**
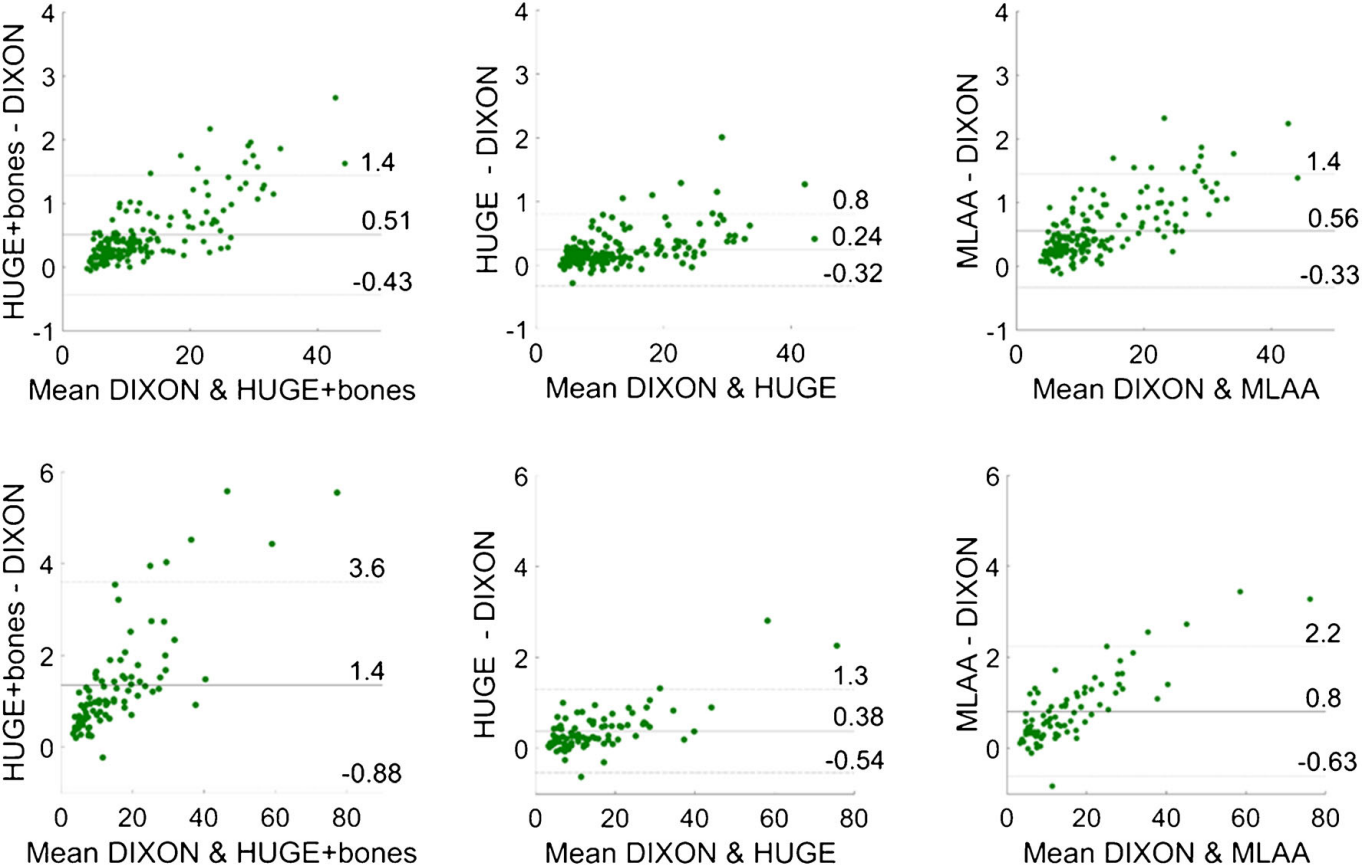
Bland-Altman plots for each of the three evaluated methods against standard Dixon: soft tissue lesions (top) and bone lesions (bottom); the agreement limits in each graph were calculated as ± 1.96 × standard deviation

The correlation between each of the three evaluated methods and the standard Dixon method was plotted both for soft tissue and bone lesions. The results are shown in Fig. 3. Additionally, Bland-Altman plots are displayed in Fig. 4, showing systematic differences between each of the three evaluated methods and the standard Dixon method.

Within the ^68^Ga-PSMA datasets, using the standard Dixon-VIBE method as reference, MRD was computed, as well as minimum and maximum relative differences. These results for soft tissue and bone lesions are shown in Table 3, respectively. The Wilcoxon signed-rank test showed statistical significance in the SUV_mean_ (*p*_soft tissue_ < 0.001; *p*_bone_ < 0.001) and SUV_max_ (*p*_soft tissue_ < 0.001; *p*_bone_ < 0.001) differences between the corresponding images as well as the corresponding background uptakes (*p*_soft tissue_ < 0.001; *p*_bone_ = 0.0014).

**Table 3:** Lesion uptakes in soft tissue (up) and bones (down)—^68^Ga-PSMA PET

Dixon w/ bone vs. Dixon w/o bone (soft tissue)					
	MRD	[MIN, MAX]	*r*	*R*^2^	*p* value
SUV_max_	2.69 ± 1.75	[− 0.31, 12.60]	0.9998	0.9973	< 0.001
SUV_mean_	2.54 ± 2.33	[− 4.05, 13.87]	0.9996	0.9964	< 0.001
BG	-	-	0.9348	0.8057	< 0.001
Dixon w/ bone vs. Dixon w/o bone (bone)					
	MRD	[MIN, MAX]	*r*	*R*^2^	*p* value
SUV_max_	8.90 ± 5.83	[1.11, 22.73]	0.9964	0.9738	< 0.001
SUV_mean_	8.96 ± 5.65	[2.06, 23.65]	0.9968	0.9738	< 0.001
BG	**-**	**-**	0.9642	0.8917	0.0014

**Fig. 5:**
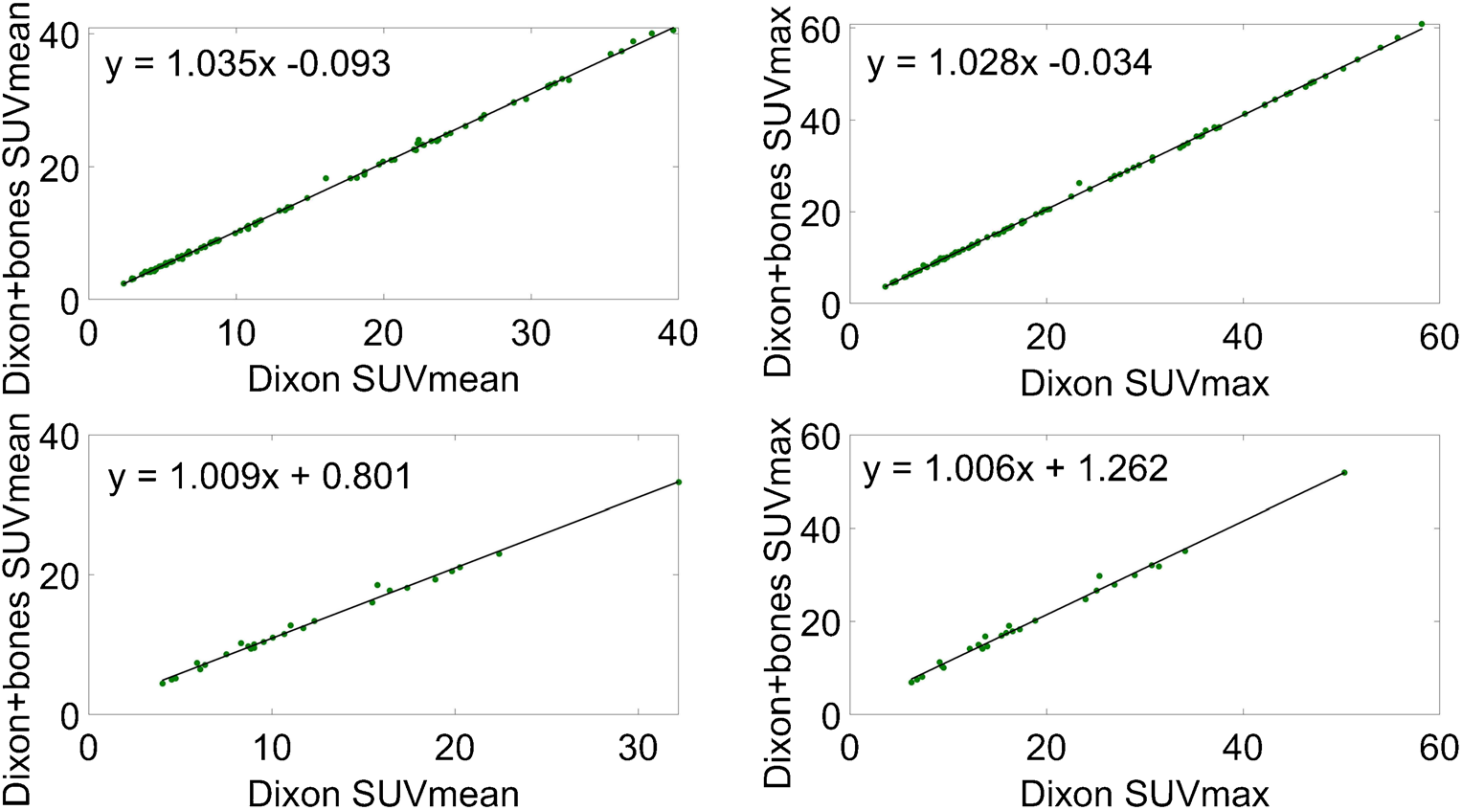
Correlation between standard Dixon with bone and Dixon without bone: soft tissue lesions (top) and bone lesions (bottom)

**Fig. 6:**
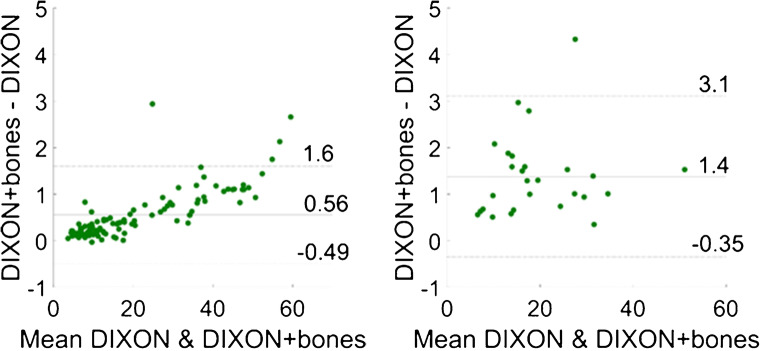
Bland-Altman plots for standard Dixon without bone and Dixon with bone: soft tissue lesions (left) and bone lesions (right); the agreement limits in each graph were calculated as ± 1.96 × standard deviation

The correlation between Dixon with bones and standard Dixon without bones is plotted in Fig. 5 both for the soft tissue lesions and for the bone lesions. In Fig. 6, Bland-Altman plots display systematic differences between each of the three evaluated methods and the standard Dixon method.

**Fig. 7:**
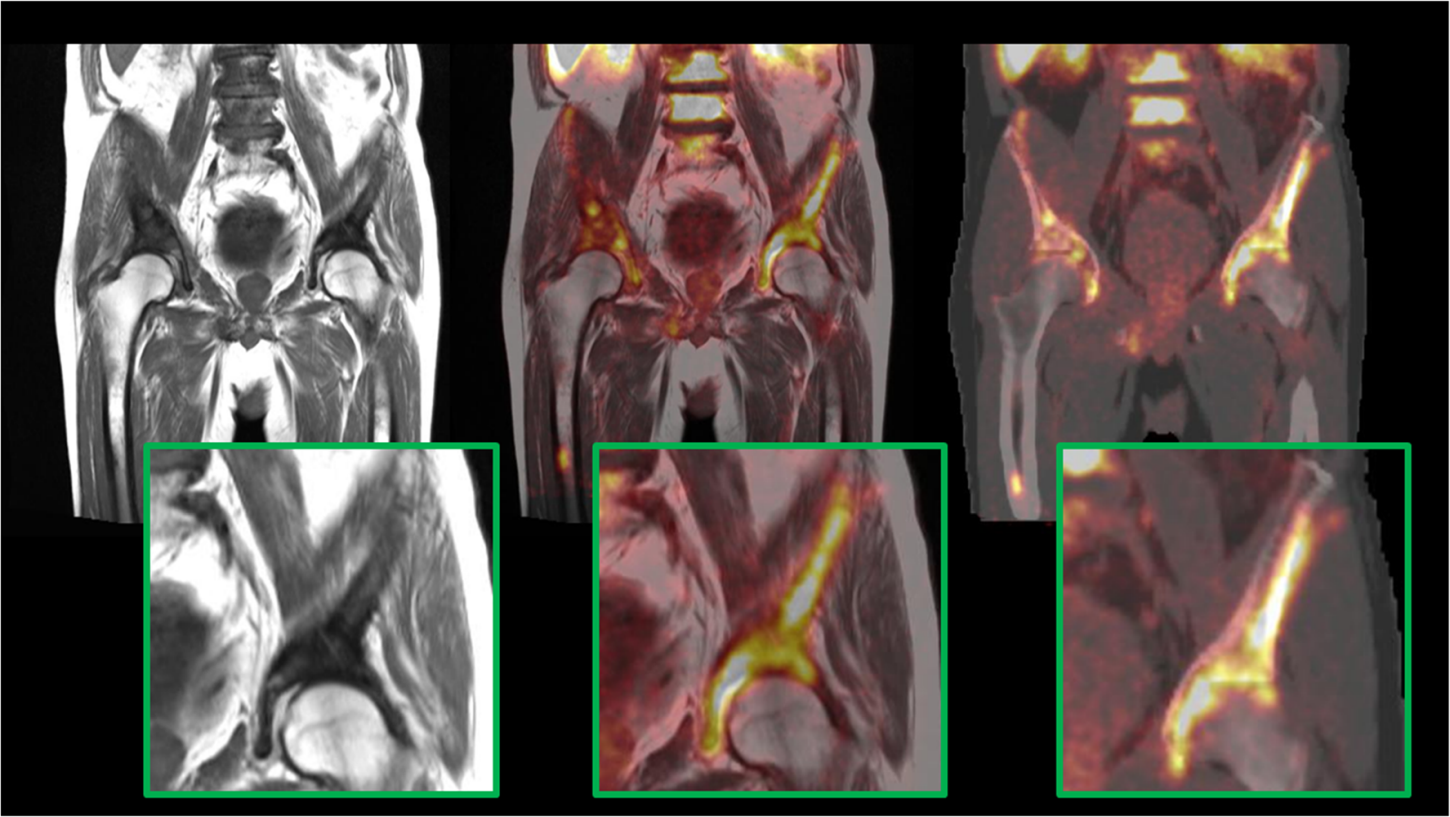
Bone registration inaccuracy in one patient: MR T1 turbo spin echo (TSE) image (left), fused MR T1 TSE with PET (middle), and fused AC map including HUGE and bones with PET (right)

Bone atlas registration accuracy issues were encountered for every case. The highest registration errors detected in the whole pelvis had a mean value of 12.5 mm (min = 2 mm, max = 32.5 mm), whereas, specifically at the femur, the highest registration error was 7.9 mm. Due to these errors, 25 analyzed lesions were affected (17 in soft tissue and 8 in bones; in total, 9.5% of all analyzed lesions) in 21 patients, encountering a MRD of 5.7% [min = 0.4%, max = 15.3%] in SUV_mean_ and 5.5% [min = 1.9%, max = 14.7%] in SUV_max_. An example of bone registration inaccuracy in one patient is seen in Fig. 7, wherein the central image, the right pubis, and ilium are delineated by the encompassing bone metastases, while on the right, the atlas bone is registered with a significant displacement with regard to the bone metastases.

## Discussion

In this study, we evaluated the effects of including bones in the AC map and extending the MR field-of-view on clinical PET/MR lesion quantification for ^68^Ga- and ^18^F-labbelled PSMA ligands and found bone atlas registration to be problematic and the overall differences introduced to be prone to biases and, on average, not affecting clinical evaluations.

Chronologically, clinical Biograph mMR PET images were first reconstructed using standard Dixon AC only, followed by Dixon extended with MLAA, and currently, since the E11 system software update, using Dixon extended with both HUGE and bones. As PET quantification is used to identify cancer therapy response, Dixon+MLAA method was included in the study to evaluate how the consecutive clinically utilized methods impacted PET quantification. This is especially important for PCa patients whose therapy response was followed over the period during which PET image quantification was affected by new AC methods. Additionally, the MLAA extension was included for comparison with the MR FoV extension using HUGE, as both methods aim for the same kind of compensation.

Obtained through the qualitative assessment of 196 reconstructed image volumes, our first finding holds that no difference in the lesion detection and qualitative analysis of those lesions could be reported. Regarding a visually more correct truncation compensation, despite one instance of partial failure, the MR FoV extension with HUGE achieved better results compared with the extension with MLAA method, providing visually more consistent LACs in the compensated regions.

The quantitative evaluation made use of SUV_max_ and SUV_mean_ values to show the effects on the tracer uptake caused by different AC generation methods. Although disputed, the SUVs are nowadays commonly used in clinical oncology and, particularly, for assessing patient response to cancer therapy. We employed them as our metric, disregarding their undocumented usefulness in this particular setting (e.g., validation only for ^18^F-FDG, correlation to Patlak plots), since our goal was to demonstrate the clinical difference from a qualitative (the accuracy of detection) and a quantitative perspective (the potential to track quantitative differences as a part of treatment monitoring). Clinically significant qualitative differences here imply any kind of lesion detection failure as well as noticeably poorer or altered lesion visualization, whereas in the quantitative context, this term suggests differences in the SUV_mean_ or SUV_max_ that lie outside of 10% from the reference value and as such may affect the PCa staging or therapy response assessments.

Based on our findings within the ^18^F-PSMA cohort, the MRD in the SUVs measured in soft tissue lesions (Table 1) did not exceed 5% for any of the evaluated methods, remaining within likely acceptable limits [23]. All three assessed methods were characterized with high standard deviation, whereby for Dixon extended with HUGE only, standard deviations went even higher than the MRD values, implying this patient cohort had featured both under- and overestimations of SUVs when compared with standard Dixon. Moreover, introducing the major bones and HUGE did not always lead to increased tracer uptake—noticeable oscillations were present. Regarding SUVs measured in bone lesions (Table 2), the highest MRD of up to 10% was found in Dixon-VIBE extended with both HUGE and bones, while for the other two investigated methods, MRD remained below 6%. Considering that similar, and occasionally higher, SUV differences are normally introduced even due to using standard scatter correction instead of an un-renormalized absolute one, these differences could also be considered within acceptable limits [24]. Additionally, one should bear in mind that even the gold standard, i.e., CT-based PET AC, can accumulate errors of up to 10% due to the effects of CT beam hardening and the resulting uptake overestimation along the bone edges [25].

Moreover, the correlation plots did not significantly vary regardless of the method tested against standard Dixon-VIBE. Both in soft tissue and bone lesions, high correlation (*r* > 0.998) was found for SUV_max_ as well as SUV_mean_. In addition to the correlation plots, Bland-Altman plots were employed to compute and depict the estimated bias and fluctuations in SUV_max_ between the compared methods. For this purpose, SUVmax was taken as it is insensitive to the ROI definition or the threshold applied; i.e., it is not subject to intra- and interobserver variability. For the soft tissue lesions, a slight positive bias was noticeable in all Bland-Altman plots (Fig. 4), albeit with noticeable fluctuations. In the case of Dixon-VIBE extended with HUGE, this bias was less pronounced (*y* = 0.24 ± 0.29), implying a negligible difference if soft tissue lesions located in the pelvis were measured by standard Dixon-VIBE or its updated version with HUGE. The other two methods, Dixon-VIBE with both HUGE and bones and Dixon-VIBE with MLAA (Fig. 4, top left and top right, respectively), showed a similar behavior between each other, depicted in their Bland-Altman plots featuring positive biases twice as high (*y* = 0.51 ± 0.45 and *y* = 0.56 ± 0.43, respectively) with increased fluctuations. In both cases, a trend can be observed, showing the bias between the tested methods increasing with the uptake of the analyzed lesion. This can be explained directly through the Beer-Lambert law:

1 $$ {I}_z={I}_0{e}^{-\mu z}, $$

where *I*_0_ and *I*_*z*_ are the initial PET signal intensity and the detected PET signal intensity after passing through a tissue of thickness *z* and LAC *μ*. Applying this law to a setting with multiple tissue types of different attenuation properties, one can deduce that the difference of the two corrected PET signals (using AC with and without bones, respectively) is directly proportional to the detected, uncorrected PET signal. Thus, higher lesion uptakes will result in higher absolute differences (shown in Bland-Altman plots) between these two corrected PET signals. These more prominent biases as well as the following fluctuations imply less agreement between the compared methods, but even as such, as discussed already, do not result in a clinically significant uptake difference measured in soft tissue lesions. In the case of the bone lesions, however, a higher bias in the Bland-Altman plot (Fig. 4, bottom) is evident primarily for Dixon-VIBE with both HUGE and bones (*y* = 1.4 ± 1.22), but also, to a lesser extent, for Dixon-VIBE with MLAA (*y* = 0.80 ± 0.71). The difference between the two biases stems from the fact that an MLAA AC map features lower LACs in certain regions in comparison with a HUGE+bone AC map. The above-mentioned regions include the arms and all the places where the MLAA map does not feature the bones present in the HUGE+bone AC map. Given the same amplitude of the uncorrected PET signal, and applying Eq. 1, the bias amplitudes are driven by the differences between the LACs present in the HUGE+bones and MLAA AC maps, respectively. Furthermore, given the nature of the MLAA method, the bias introduced here is also object-dependent.

Our findings within the ^68^Ga-PSMA dataset (Table 3) support the above-discussed results acquired with ^18^F-PSMA. In the soft tissue lesions, MRD did not exceed 3%, whereas in the bone lesions, MRD was not higher than 9%. The correlation between standard Dixon-VIBE and Dixon with bones proved to be very high, taking all lesions into account. In the case of soft tissue lesions, the Bland-Altman plot showed a low bias between the two compared methods (*y* = 0.56 ± 0.53), while for bone lesions, it was significantly higher (*y* = 1.4 ± 0.87), albeit with no visible trends and within its respective agreement limits. However, given that we analyzed 27 bone lesions in our ^68^Ga-PSMA patient dataset, these results could be limited by a small sample size.

The bone registration issues should also be considered. Registration errors of up to 32.5 mm and bones missing from 8 AC maps introduce additional uncertainty in the quantification accuracy, especially for lesions adjacent to or at the very edge of bones. Adding bones inside the body with such an atlas, hence, proves to be more complicated and prone to error as compared with the atlases of the head.

Finally, this study has certainly its limitations: in the absence of the absolute ground truth data, all results were compared using linear regression against the standard Dixon-VIBE method and, hence, all reported differences for each evaluated method were using this method as reference.

## Conclusion

In conclusion, the addition of the evaluated, more complex AC schemes did not produce clinically significant changes in quantifying lesions in PSMA-ligand PET imaging compared with the version introduced with PET/MR initially. Taking into account the occurring inaccuracy that often follows using a bone atlas in whole-body imaging, as well as the similar magnitude of PET quantification biases easily introduced by other mentioned PET correction algorithms, mean tracer uptake differences of 4% and 10% for pelvic soft tissue and bone lesions, respectively, can be regarded as differences within the likely acceptable limits. Lesion visibility was not affected either. We therefore conclude that compensating for truncations in the arms and including major bones in the PET AC did not produce clinically significant differences in PSMA diagnostic accuracy and it allows for serial studies without the need of reprocessing. Using atlas-based methods when comparing serial scans for bone lesions is, nevertheless, not advisable without great caution in the absence of ground truth data.

## Data Availability

The datasets generated and/or analyzed during the current study are not publicly available as they contain information that could compromise patients’ privacy but are available from the corresponding author on reasonable request and in anonymous form.

## Abbreviations

PET: Positron emission tomography
MR: Magnetic resonance
AC: Attenuation correction
HUGE: *B*_0_ (static magnetic field) homogenization using gradient enhancement
PCa: Prostate cancer
PSMA: Prostate-specific membrane antigen
MLAA: Maximum-likelihood reconstruction of attenuation and activity
SUV: Standardized uptake value
FoV: Field of view
CT: Computed tomography
LAC: Linear attenuation coefficient
UTE: Ultra-short echo time (magnetic resonance sequence)
ZTE: Zero echo time (magnetic resonance sequence)
SNR: Signal-to-noise ratio
FDG: Fluorodeoxyglucose
mpMRI: Multiparametric magnetic resonance imaging
BMI: Body mass index
CAIPIRINHA: Controlled aliasing in parallel imaging results in higher acceleration
OP-OSEM: Ordinary Poisson ordered-subset-expectation maximization
FWHM: Full width at half maximum
VIBE: Volumetric interpolated breath-hold examination
PERCIST: Positron emission tomography response criteria in solid tumors
MRD: Mean relative difference
